# Supplementary figures and images for: Patterns of telomere length with age in African mole-rats: New insights from quantitative fluorescence in situ hybridisation (qFISH)
Source: PeerJ. 2020 Dec 4;8:e10498. doi: 10.7717/peerj.10498 (PMC7720729; doi:10.7717/peerj.10498)

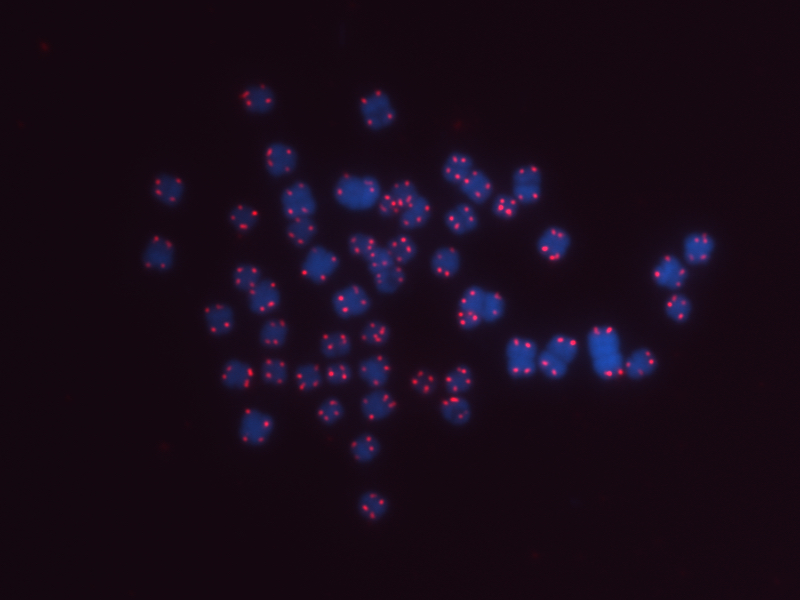

Supplement: Supplemental Information 1 — DNA is stained blue (with DAPI) and telomeres in red (with Cy3 PNA probe). 2n=53, as reported for males in other Cryptomys species (2n=54 for females due to a Y-chromosome-autosome translocation in males; Deuve et al., 2007). [file peerj-08-10498-s001.jpg]

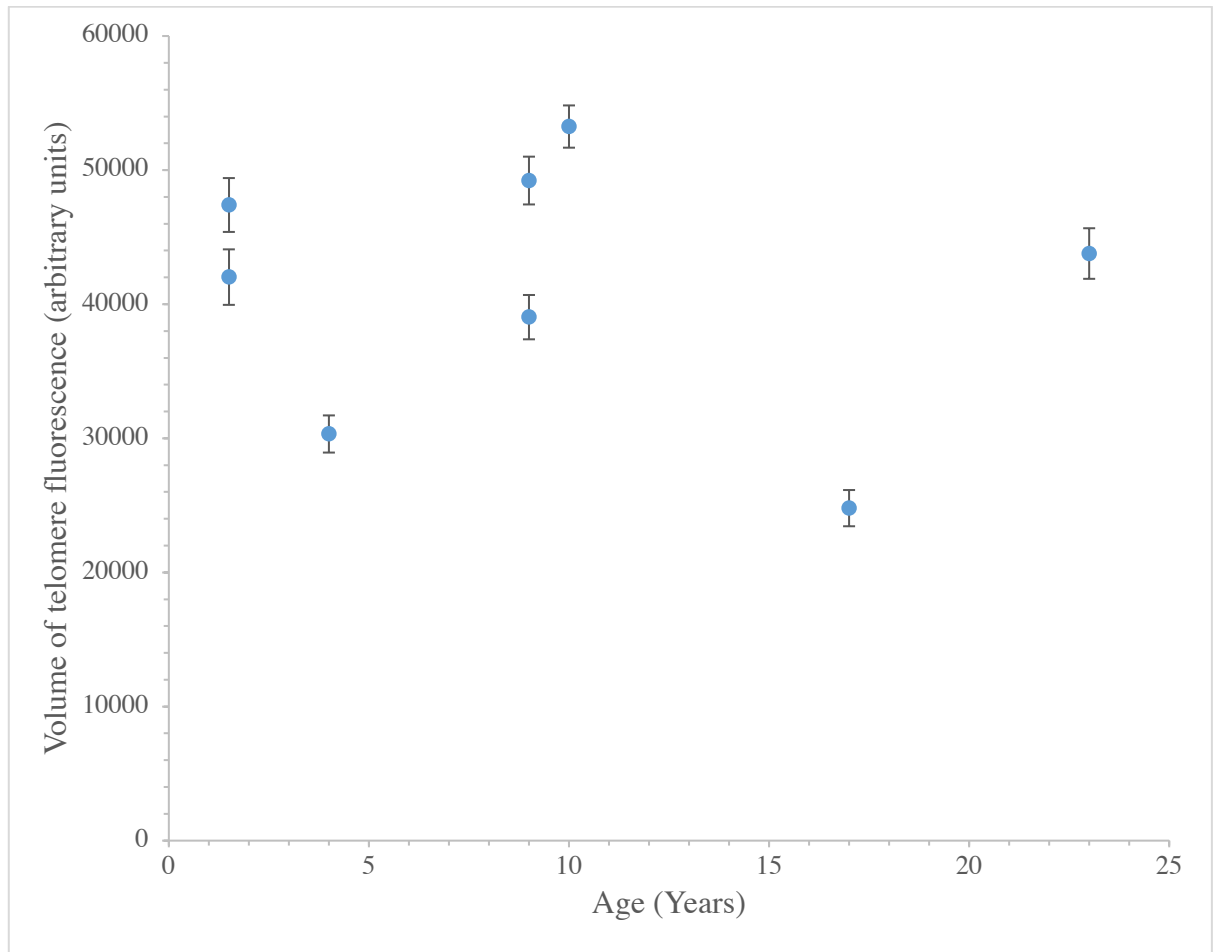

Supplement: Supplemental Information 2 — Scatter plot of mean ± SEM telomere size variation (expressed as volume of telomere fluorescence) with age for the naked mole-rats show in Figure 2. Points correspond to an individual animal (n=50 cells counted in each). [file peerj-08-10498-s002.pdf]
