# Supplementary material for: Patterns of telomere length with age in African mole-rats: New insights from quantitative fluorescence in situ hybridisation (qFISH)
Source: PeerJ. 2020 Dec 4;8:e10498. doi: 10.7717/peerj.10498 (PMC7720729; doi:10.7717/peerj.10498)
Supplement: Supplemental Information 4 — Age class based on molar tooth eruption and wear for DMR and MMR (Bennett, Jarvis & Wallace, 1990), or absolute age in years for NMR. Age category as described in methods. Shaded rows correspond to NMR samples with too few cells to count, not included in the final analysis. [file peerj-08-10498-s004.pdf]

Species	Individual	Sex	Age class	Age category
DMR	2.2	Female	5	Old
DMR	2.5	Female	5	Old
DMR	2.6	Female	5	Old
DMR	8.4	Male	5	Old
DMR	8.5	Male	5	Old
DMR	9.11	Female	1	Young
DMR	9.13	Female	5	Old
DMR	9.5	Female	1	Young
DMR	11.6	Female	1	Young
DMR	11.9	Male	1	Young
DMR	11.10	Male	1	Young
DMR	11.11	Female	1	Young
MMR	7.1	Female	2	Middle-aged
MMR	7.2	Female	2	Middle-aged
MMR	10.1	Female	4	Old
MMR	12.1	Male	4	Old
MMR	12.2	Male	4	Old
MMR	12.3	Male	2	Middle-aged
MMR	12.4	Female	4	Old
MMR	12.5	Male	3	Middle-aged
MMR	12.6	Female	3	Middle-aged
MMR	12.7	Female	3	Middle-aged
MMR	12.8	Female	3	Middle-aged
MMR	12.9	Male	3	Middle-aged
MMR	12.11	Female	2	Middle-aged
MMR	12.12	Male	2	Middle-aged
MMR	18.1	Female	1	Young
MMR	18.2	Female	1	Young
MMR	18.3	Female	1	Young
MMR	18.4	Male	3	Middle-aged
MMR	18.5	Male	3	Middle-aged

MMR	18.6	Male	3	Middle-aged
MMR	18.7	Female	2	Middle-aged
MMR	19.1	Female	4	Old
MMR	22.2	Male	1	Young
MMR	23.1	Male	1	Young
MMR	23.2	Male	1	Young
MMR	23.3	Male	1	Young
MMR	30.1	Female	1	Young
NMR	F535	Female	23	Old
NMR	F798	Female	4	Young
NMR	F894	Female	17	Old
NMR	M1	Male	1.5	Young
NMR	M2	Male	1.5	Young
NMR	F048	Male	9	Middle-aged
NMR	F775	Male	9	Middle-aged
NMR	F791	Female	22	Old
NMR	M825	Male	2	Young
NMR	M781	Male	2	Young
NMR	M124	Male	10	Middle-aged
NMR	M353	Male	10	Middle-aged
NMR	F10	Female	20	Old

Bennett NC, Jarvis JUM, Wallace DB. 1990. The relative age structure and body masses of complete wild-captured colonies of two social mole-rats, the common mole-rat, *Cryptomys hottentotus hottentotus* and the Damaraland mole-rat, *Cryptomys damarensis*. *Journal of Zoology* **220:**469–485 DOI:10.1111/j.1469-7998.1990.tb04319.x.
